# X-ray Ptychographic Imaging and Spectroscopic Studies of Plasma-Treated Plastic Films

**DOI:** 10.3390/polym14132528

**Published:** 2022-06-21

**Authors:** Mehdi Ravandeh, Masoud Mehrjoo, Konstantin Kharitonov, Jan Schäfer, Antje Quade, Bruno Honnorat, Mabel Ruiz-Lopez, Barbara Keitel, Svea Kreis, Rui Pan, Seung-gi Gang, Kristian Wende, Elke Plönjes

**Affiliations:** 1 Leibniz Institute for Plasma Science and Technology, Felix-Hausdorff-Straße 2, 17489 Greifswald, Germany; mehdi.ravandeh@inp-greifswald.de (M.R.); jschaefer@inp-greifswald.de (J.S.); quade@inp-greifswald.de (A.Q.); bruno.honnorat@inp-greifswald.de (B.H.); 2 Deutsches Elektronen-Synchrotron DESY, Notkestraße 85, 22607 Hamburg, Germany; konstantin.kharitonov@desy.de (K.K.); mabel.ruiz-lopez@desy.de (M.R.-L.); barbara.keitel@desy.de (B.K.); svea.kreis@desy.de (S.K.); rui.pan@desy.de (R.P.); seung-gi.gang@desy.de (S.-g.G.); elke.ploenjes@desy.de (E.P.)

**Keywords:** corona discharge, polyethylene terephthalate, scanning electron microscopy, free electron laser, X-ray photoelectron spectroscopy

## Abstract

Polyethylene terephthalate (PET) is a thermoplastic polyester with numerous applications in industry. However, it requires surface modification on an industrial scale for printing and coating processes and plasma treatment is one of the most commonly used techniques to increase the hydrophilicity of the PET films. Systematic improvement of the surface modification by adaption of the plasma process can be aided by a comprehensive understanding of the surface morphology and chemistry. However, imaging large surface areas (tens of microns) with a resolution that allows understanding the surface quality and modification is challenging. As a proof-of-principle, plasma-treated PET films were used to demonstrate the capabilities of X-ray ptychography, currently under development at the soft X-ray free-electron laser FLASH at DESY, for imaging macroscopic samples. In combination with scanning electron microscopy (SEM), this new technique was used to study the effects of different plasma treatment processes on PET plastic films. The studies on the surface morphology were complemented by investigations of the surface chemistry using X-ray photoelectron spectroscopy (XPS) and Fourier transform infrared spectroscopy (FT-IR). While both imaging techniques consistently showed an increase in roughness and change in morphology of the PET films after plasma treatment, X-ray ptychography can provide additional information on the three-dimensional morphology of the surface. At the same time, the chemical analysis shows an increase in the oxygen content and polarity of the surface without significant damage to the polymer, which is important for printing and coating processes.

## 1. Introduction

Plastics are notoriously difficult to bond because of their low surface energy, which reduces wetting of the surface by the adhesive during application, resulting in a poor bond [1]. Poly (ethylene terephthalate) (PET) is one of the most widely used plastics in industry because of its high hardness, strength, thermal stability, chemical resistance, and formability [2]. However, the hydrophobic nature of PET can be disadvantageous for applications such as adhesion, painting, printing, metallization, and so on [2]. Therefore, chemical [3], enzymatic [4] and physical modifications have been carried out to make the PET surface more hydrophilic. There are several methods to treat polymeric surfaces, such as wet chemistry, UV irradiation, corona discharge, flame treatment and ozone treatment [4,5,6,7,8]. Fundamental and applied research on these processing methods has been conducted in recent decades [2,3,7,9] to study physical and chemical modification processes on the polymer surface.

Due to cost-effectiveness and ease of use, corona treatment is probably the most commonly used industrial treatment for polymer films [10,11]. In an industrial corona treatment, a dielectric barrier discharge occurs when a high voltage is applied to an electrode and a grounded backup roller to cause ionization of the air. The plastic film is introduced into the gap between the electrode and the backing roll, and when its surface is bombarded with high-speed electrons and reactive species, the molecular bonds on the surface of most polymer substrates are cleaved and modified [12]. The corona derived reactive oxygen species which include ozone, atomic oxygen, superoxide anion radical (O_2_^−^), singlet oxygen (^1^O_2_), hydroxyl radical (**^·^**OH) and hydroperoxyl radical (HO_2_**^·^**) [13], introduce new functional groups at the surface of polymer films. Hydroxyl, carbonyl, carboxyl and ester groups are most commonly found on the surfaces of polymers after plasma treatments, which increases the surface energy and improves the wetting and adhesion properties of polymer films [14].

Various techniques are used to characterize the surface morphology such as atomic force microscopy (AFM) and scanning electron microscopy (SEM). The surface chemistry of the polymer films can be investigated using X-ray photoelectron spectroscopy (XPS), Fourier-transform infrared spectroscopy (FT-IR), and Raman spectroscopy [9,15]. Furthermore, the surface-wetting of the films is characterized by contact-angle measurements [16]. The desire to probe the three-dimensional structure of material surfaces of millimeter or even centimeter size at high resolution for a wide range of applications in materials characterization has motivated a continuous and long-lasting effort to develop suitable X-ray imaging techniques [17,18]. X-ray ptychography has a unique advantage in achieving this goal due to its lens-less concept allowing high-resolution imaging which is solely limited by the wavelength of the light source [18]. It is a scanning coherent diffraction imaging (CDI) approach that enables lens-less imaging of extended samples in the X-ray regime [19] due to its scanning nature [18,20]. It has several advantages over AFM. First, it has higher throughput (several mm^2^/h) with simultaneously compatible (tens-hundreds nm) spatial resolution allowing for faster measurements of extended samples. Additionally, ptychography inflicts less sample damage and does not charge the samples, thus being more appropriate for the imaging of sensitive or insulating samples [21]. Moreover, contrary to AFM, the image contrast in ptychography not only originates from height differences but also from the variations of the sample’s electron density thus allowing chemically sensitive imaging. An obvious disadvantage, however, is the need for a free electron laser (FEL) or a synchrotron source, i.e., a large-scale accelerator-based research infrastructure, rather than a laboratory instrument. Thus, the techniques should be seen as complementary.

In ptychography, a sample is densely scanned with consecutive X-ray pulses, i.e., the scan positions overlap each other, and a series of diffraction patterns is recorded. The diffraction patterns are computationally analysed using ptychographic algorithms [22,23] such as the classical ePIE algorithm [24] the mixed state ptychography [25], and, more recently, the modern adaptive automatic differentiation (A-AD)-powered ptychography [26] used in this work. The application of ptychography has recently gained momentum with the use of X-ray free-electron lasers (FELs) due to their unprecedentedly intense and coherent photon pulses [27]. 

In this article, we report on the first application of X-ray ptychography imaging on industrially relevant samples performed at FLASH, the Free-electron LASer in Hamburg at the Deutsches Electron-Synchrotron DESY [28,29]. Recent computational advances to introduce a novel ptychography algorithm based on adaptive automatic differentiation (A-AD) [26,30] have paved the way to perform high-resolution ptychographic imaging at FLASH. The technique is used to study the morphological changes on PET films treated with two different types of plasma surface treatment. The results are compared to SEM images of the samples and complemented with studies on the surface chemistry of the PET films using XPS and attenuated total reflection- infrared spectrometry (ATR-IR) techniques.

## 2. Materials and Methods

### 2.1. Plasma Treatments 

**Figure 1:**
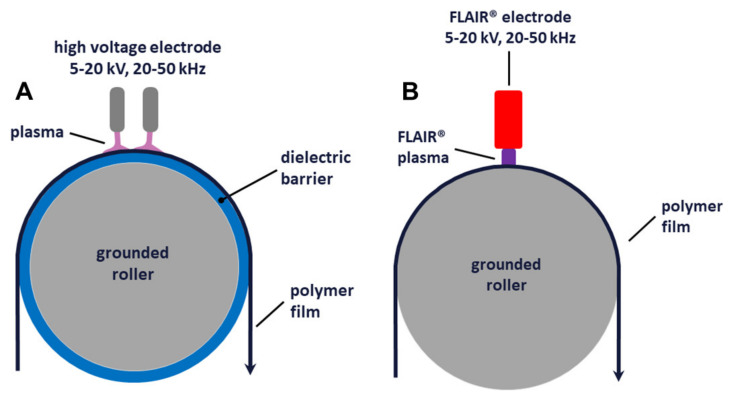
Schematic sketch of the setup for corona (**A**) and FLAIR^®^ (**B**) treatments of PET films. The produced plasma is shown in light purple for the Corona treatment and in purple for the FLAIR^®^ treatment. The power density of 200 W/cm^2^ and 5 W/cm^2^ and treatments time of 0.09 s and 3.6 s were used for FLAIR^®^ and corona treatments, respectively. The total energy per area of the plasma-treated film was kept the same at 180 kJ/m^2^ for both treatments.

Samples of BOPET (Biaxially Oriented Poly (Ethylene Terephthalate) Films of Type Mylar CW02 (Provided by DuPont Teijin Films Luxembourg, Luxembourg) in a size of 21.0 × 29.7 cm^2^ and with 1.4 µm thickness were treated with atmospheric pressure plasmas in the industrial corona and FLAIR^®^ treatment systems from Plasmawerk Hamburg GmbH (Hamburg, Germany). In industrial surface treatment, corona treatment is an atmospheric pressure plasma technology using a medium frequency (typically between 20 kHz and 50 kHz) dielectric barrier discharge in ambient air. Such systems are widely used in the polymer extrusion and processing industry and can be scaled up for continuous plasma treatment of polymer films up to 10 m wide and at speeds greater than 1000 m/min. Corona treatment equipment for continuous treatment of polymer films (see Figure 1A) consists of an electrically grounded roller supporting the moving film and a high voltage electrode placed at a distance of 1–2 mm from the roller. Either the roller, the electrode, or both require a dielectric coating. Often a 2 mm to 4 mm thick silicone rubber on the roller or a 1 mm to 3 mm thick Al_2_O_3_ ceramic on the roller or electrode is used. Applying a high voltage of the order of 10 kV to the electrode creates a plasma in the air gap between the electrode and the roller. The polymer film passing through the air gap is plasma treated on the side facing the electrode.

In a FLAIR^®^ treatment unit (see Figure 1B), the general setup is similar to that of a corona treatment unit, with a film supporting grounded roller and a high-voltage electrode a few mm apart. While the power density of the plasma generated between the high-voltage electrode and the roller in an industrial corona treater is limited to a few W/cm^2^ and almost never exceeds 8 W/cm^2^, FLAIR^®^ treaters operate with power densities of up to several 100 W/cm^2^. In this power density range, the plasma species in the air plasma differ significantly from those in a corona treatment plasma and different effects on the morphology and chemical composition of the polymer film can be expected. For this study, BOPET film samples were treated by corona and FLAIR^®^ plasma at a power density of 5 W/cm^2^ and 200 W/cm^2^, respectively. In both cases, the total energy per area of the plasma-treated film was kept the same at 180 kJ/m^2^. This was achieved by varying the speed of the film and thus the treatment time: 3.6 s in the case of corona and 0.09 s in the case of FLAIR^®^. Both treatment units are shown schematically in Figure 1. These identical energies per area were chosen to ensure comparability between the samples. The energy per area applied in this study is higher than the energy used in industrial processing to ensure a significant and measurable morphological change on the film surface as a test sample for X-ray ptychography.

### 2.2. X-ray Ptychography

**Figure 2:**
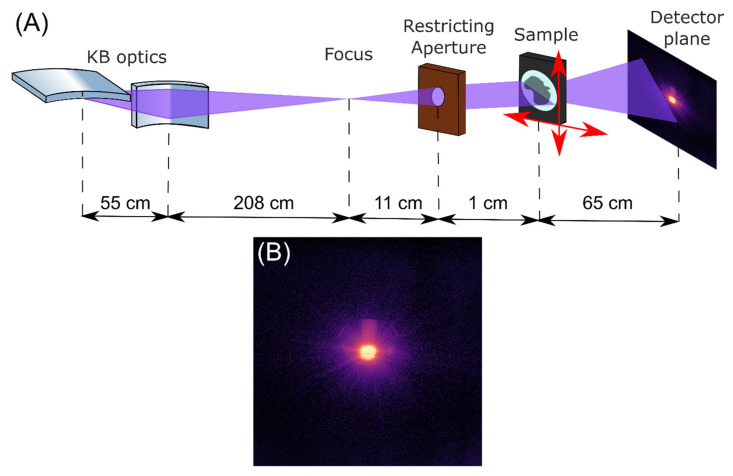
Ptychography experiment setup (**A**) and a typically measured diffraction (13 × 13 mm^2^) pattern (**B**).

The surface morphology of the sample specimen was studied using X-ray ptychography imaging at FLASH, the Free-electron LASer in Hamburg, at DESY, the German Electron Synchrotron (Hamburg, Germany). This accelerator-based light source for the soft X-ray range provides pulses at wavelengths from 4–90 nm (fundamental) with femtosecond pulses and exceptionally high photon pulse energies [27]. The experiment was performed at the beamline FL24 at FLASH2 at a wavelength of λ = 2.66 nm (third harmonic of 7.8 nm) corresponding to E_photon_ = 466 eV with an average photon pulse energy of E_pulse_ = 90 µJ. The experimental setup is shown in Figure 2. The FEL beam was focused using a pair of bendable Kirkpatrick–Baez (KB) mirrors (FERMI, Trieste, Italy) [27,31] and confined with the restricting aperture. An ANDOR iKON-M SO CCD camera (1024 × 1024 pixels, 13 × 13 µm^2^ each, Oxford Instruments Andor, Tokyo, Japan) was used to record the diffraction patterns. 

The interaction between the sample and the X-ray radiation results in modulation of the incoming radiation wavefield ψ_in_ by the sample O_(x,y)_ that can be expressed as [32]:

ψ_out_ = ψ_in_ · O_x,y_ = ψ_in_ · [exp (−kβ_eff(x,y)_) exp (−ikδ_eff(x,y)_)] (1)

where β_eff(x,y)_ = βz_(x,y)_, δ_eff(x,y)_ = δz_(x,y)_ are the effective refraction index components of the sample, z_(x,y)_ is the 2D sample thickness distribution, and k is the wave vector defined by the wavelength of λ as 2π/λ. By ptychography measurements, the effects of the sample modulation function O_(x,y)_ are reconstructed for all scan positions, providing information about the morphological properties of the sample. 

Ten diffraction patterns were measured at each scan position with a frame rate of 1 Hz, scanning a sampling grid of 20 × 20 positions with the 90% overlap, i.e., a sampling area of 145 × 145 µm^2^. The most intense diffraction pattern was selected as representative for each of the positions for the ptychography reconstruction to minimize the influence of the fluctuations typical for FELs. A low heat load on the sample was ensured and saturation of the CCD camera was prevented by reducing the total intensity of the FEL by employing metallic foil filters and a gas-based attenuator [33].

To reconstruct the surface morphology of the samples, an automatic differentiation ptychography algorithm was developed [26]. This ptychography algorithm has been devised to allow an analysis of the measured diffraction patterns despite the fact that FELs based on the self-amplified spontaneous emission process (SASE) [34] inherently exhibit pulse-to-pulse fluctuations of intensity, spectrum and pointing and have only a partial spatial coherence (typically 60–80%). At the same time, scanning imprecisions can be numerically integrated into the algorithm. The computational power of the A-AD routine to analyse the measured data provides very high flexibility for performing novel ptychographic imaging at FELs. In [26] a detailed description of its use with the measurements at FLASH can be found.

The entire reconstruction included 300 iterations of the A-AD ptychography algorithm. We utilized 4 GPU NVIDIA P100 in parallel for the computations, which resulted in an iteration time of 1 s with a total surface morphology reconstruction time of 5 min. The resolution of the reconstruction was estimated by calculating the Fourier ring correlation between two independent reconstructions [35].

### 2.3. Scanning Electron Microscopy (SEM)

The PET samples have been investigated using a high-resolution scanning electron microscope SEM JSM-7500F (JEOL, Tokyo, Japan). The SEM uses field emission electron gun (FEG) and detectors for both low-angle backscattered electrons (LABE) and secondary emitted (SE) electrons, LABE and SE in-lens, respectively. The maximum spatial resolution is specified with 1.0 nm. The microscope allows to observe also non-conductive samples without any surface modifications changing the conductivity or structure of the sample surfaces. The gentle beam mode (GB high) has been applied for several samples before and after plasma treatments. In this study, the operational conditions were set as following: accelerating voltage of the primary electron beam 500 V and 1 kV, magnification up to 10,000×, working distances from 4.3 to 8 mm.

### 2.4. X-ray Photoelectron Spectroscopy (XPS)

The elemental surface composition and chemical modification of the plasma-treated samples were analysed by X-ray photoelectron spectroscopy (XPS) using an AXIS Supra spectrometer (Kratos Analytical, Manchester, UK). Spectra were acquired using monochromatic X-rays, Al kα (15 kV, 10 mA for general spectra and 15 kV, 15 mA for highly resolved measured C 1s peaks), with a medium magnification lens (field of view 2) by selecting the slot mode. Survey spectra were recorded at a pass energy of 160 eV. A pass energy of 80 eV was used to estimate the chemical elemental composition and 10 eV was used for the high-resolution C 1s peaks to study the chemical functional groups. Charge neutralization was performed for all samples to reduce possible differential charge effects. 

Data processing was performed using CasaXPS software, version 2.15 (Casa Software Ltd., Teignmouth, UK). After subtraction of a Shirley background, peaks were quantified using a Gauss–Lorentzian GL(30) peak shape. For quantification, an average of the data measured at three different sample points was calculated [36].

### 2.5. Attenuated Total Reflection-Infrared Spectrometry (ATR-IR)

The infrared spectra were acquired with a Fourier Transform Infrared (FT-IR) spectrometer Vertex 70 v (Bruker, Leipzig, Germany) using the MCT mid-band detector D316025. The spectra were obtained using a 20× germanium ATR objective mounted on a Hyperion 3000 microscope (Bruker, Leipzig, Germany) by pressing the objective onto the surface of the PET foil which was placed onto the surface of a gold substrate. The contact pressure between the germanium crystal and the surface of the PET foil was >0.5 N (pressure level 2 of the ATR objective). The spectral resolution was 4 cm^−1^.The absorbance spectra were registered in the range of wavenumber 600–4000 cm^−1^. The spectra were corrected by subtracting a constant offset equal to the mean-value of the absorbance in the wavenumber range 3150–3200 cm^−1^. Each spectrum was normalized by dividing it by the maximum absorbance of the first peak located in the wavenumber range 690–790 cm^−1^. The results are an average of 8 spectra for each sample.

## 3. Results and Discussion

A comparison of X-ray ptychography imaging with scanning electron microscopy quantifies the potential of ptychography to analyse complex surfaces such as the modifications exhibited by the PET films after plasma surface treatment. Moreover, it provides additional information on the three-dimensional morphology of the surface compared to SEM. A chemical analysis of the PET films sheds further light on the effect of different types of plasma treatment on the surface chemistry of the films. 

### 3.1. Surface Morphology of Plasma-Treated PET Films

**Figure 3:**
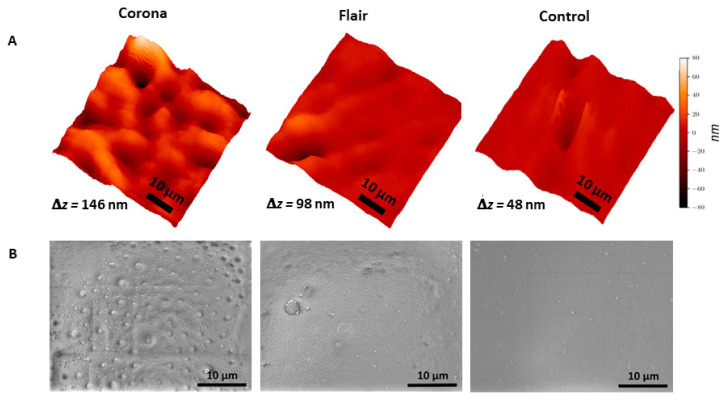
X-ray ptychography (**A**) and SEM (**B**) images of plasma-treated and control PET films. Δ*z* is the peak-to-valley difference.

Using the X-ray ptychography described in Section 2.2, surface images of the treated and untreated samples were reconstructed. A region of interest (ROI) of 50 × 50 µm^2^ of each sample with a resolution of 600 nm is shown in Figure 3A. Thus, the region of interest (ROI) may be indicative of a microscale morphologic area. The main purpose of this study is to evaluate the potential of X-ray ptychography as a method to create 3D images of complex surface profiles and evaluate their morphology. As shown in Figure 3A and Table 1, significant changes are visible between the three samples under study, which proves that this method has significant potential for the analysis of complex surfaces.

To facilitate a quantitative evaluation of the morphological changes created by the respective plasma treatments and imaged using X-ray ptychography, roughness parameters such as the root-mean-square (*RMS*) roughness *R_q_*, interfacial development *S_dr_*, kurtosis *R_ku_* and skewness *R_sk_* were calculated and tabulated in Table 1. A detailed definition of parameters and analytical expressions is given elsewhere [37].

The average roughness *R_q_* increased significantly after the plasma treatments, for corona treatment by a factor of 5, for FLAIR^®^ by nearly a factor of 2. Moreover, the interfacial development *S_dr_* and the kurtosis *R_ku_* clearly indicate the formation of a more pronounced spatial complexity by the corona treatment, while more moderate morphological modifications were produced by the FLAIR^®^ treatment. Both treated surface profiles show negative values for skewness *R_sk_* indicating predominantly valley formation, somewhat more pronounced for the FLAIR^®^ treated surface. In conclusion, the FLAIR^®^ treatment mainly introduced pronounced valley formation whereas the corona treatment modified the surface profile towards both rounded hills and valleys. 

In addition, morphological changes in ptychography images are compared to SEM images (Figure 3). Both techniques showed nearly constant morphological changes after corona treatment (peaks and valleys), but moderate surface modification with fewer, but larger, valleys in the FLAIR^®^ treated PET films. These morphological changes may be due to the breakup and removal of the top layer of the polymer film caused by interaction with the reactive species generated by the plasma on the surface. Moreover, there are some bulges at the surface of PET in SEM images that could represent the low-molecular weight oxidizing products which were reported previously after plasma treatments of polymer films [38,39]. In the literature, several studies visualized the morphological changes of PET film after plasma treatments by atomic force microscopy (AFM) [40,41,42]. In most cases, the calculated RMS parameters were obtained from AFM images of sample areas of 1 to 10 µm^2^, while X-ray ptychography allowed us to study a much larger area of the sample surface of 50 µm^2^, potentially leading to a more representative analysis of the entire treated surface. This highlights the potential of X-ray ptychography imaging for the investigation of larger surface areas within shorter measurement times compared to AFM, which is time-consuming and limited to smaller surface areas [40].

### 3.2. Surface Chemistry of Plasma Treated PET Films

**Figure 4:**
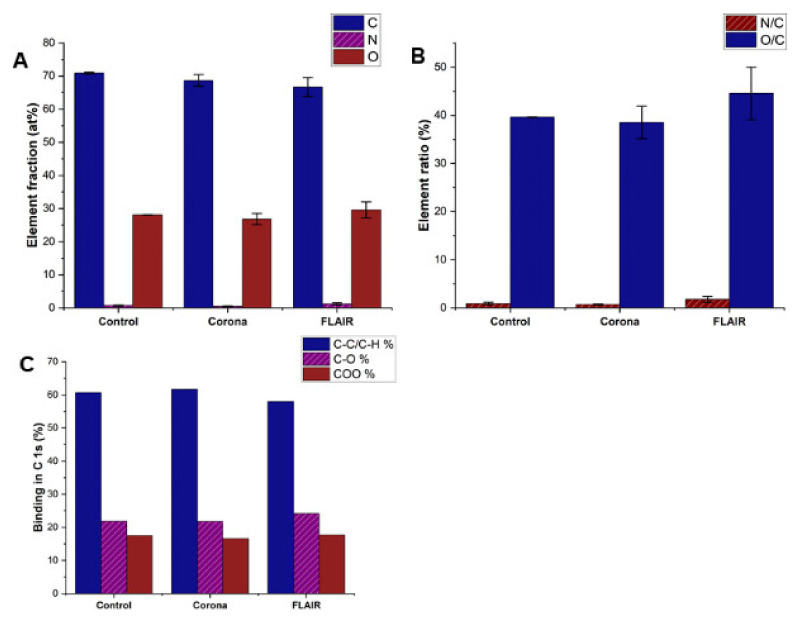
XPS results of plasma treated and control PET samples: (**A**) Elemental fractions (at %), (**B**) Elemental ratio (%), and (**C**) Binding in C 1s (%).

XPS is a powerful technique with information depths of 5–7 nm for the detection of variations in chemical composition and oxidation state. Subtle changes in peak positions and shape can yield information on changes in surface chemistry [43,44]. The XPS profiles of the counts per second (CPS) vs. binding energy (B.E.), survey and C 1s spectra for control and plasma treated PET films are shown in Figure S1. The XPS analysis shows that elemental fractions were changed after the different plasma treatments of the samples (Figure 4A), and this was more pronounced after FLAIR^®^ treatment. A 4% decrease in carbon, 1.5% increase in oxygen and 0.5% increase in nitrogen were measured for the FLAIR^®^ treated surface compared to the untreated control samples, which indicates that reactive oxygen and nitrogen species in the plasma modified the surface chemistry of the PET film. Moreover, the elemental ratios (Figure 4B) confirm this modification after FLAIR^®^ treatment, in which N/C-and O/C-ratios were very similar (0.9% and 39%) for control and corona, while a significant increase is seen in FLAIR^®^ treatment, with N/C: 1.8% and O/C: 45%. This is confirmed by the high-resolution C 1s spectra (Figure 4C), which show three different contributions at 284.7, 286.2, and 288.7 eV, corresponding respectively to the C−C and C−H of the phenyl ring, to the C−O, and to the O−C = O bonds. The increase of the C−O and O−C = O bonds and 5% drop in C−C/C−H_atom_ observed in the FLAIR^®^ treated PET films confirmed the higher degree of oxidation and the exposure of new carboxyl groups on the surface of the PET film after the FLAIR^®^ treatment compared to the corona treatment. Compared to other plastics, PET contains a relatively large amount of oxygen. A further increase in the oxygen concentration after plasma treatment would mean an attack on the aromatic cycles, which are, however, chemically relatively stable. Thus, plasma treatment is expressed in terms of rearrangement of oxygen-containing functional groups rather than a quantitative increase in the oxygen content of the material. In addition, it is conceivable that a reorientation of the surface layer and a migration of the polymer chains from the bulk to the surface has occurred within the polymer surface. Such an effect has also already been reported [45]. This could be the reason why there is no significant increase in O 1s after corona treatment compared to control.

For the same treatment energy per surface area, FLAIR^®^ produces more of the desired chemical modification on the surface while at the same time producing a lesser morphological change, which would be consistent with less chemical break-up of the surface polymer. This offers the potential for energy saving with FLAIR^®^ surface treatment instead of traditional corona treatment when targeting a certain level of chemical modification.

**Figure 5:**
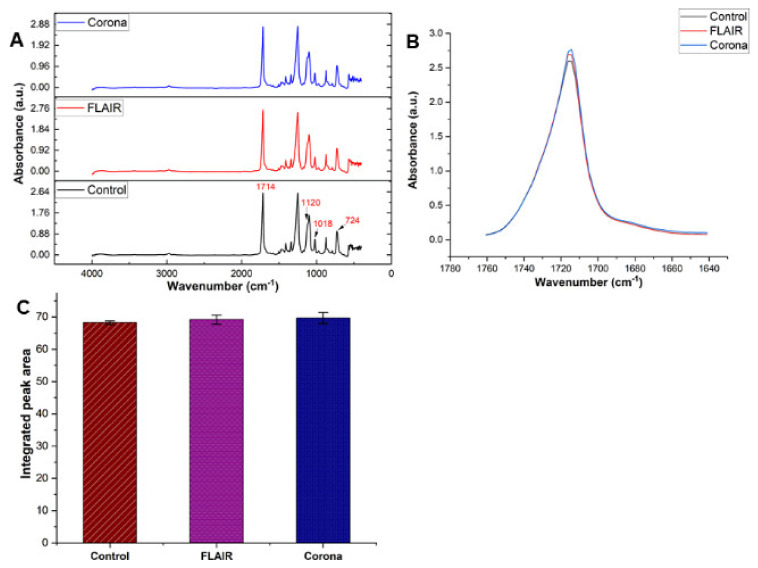
ATR-IR results of plasma-treated and untreated PET films: (**A**) Full IR spectra for the three samples, (**B**) Carbonyl region spectra (1650–1750 cm^−1^), and (**C**) Integrated peak area of carbonyl region.

In addition, the changes in functional groups after plasma treatment of the PET films were studied by Attenuated Total Reflection-Infrared spectrometry (ATR-IR). The penetration depth of ATR-IR is ~1–4 µm and the absorbance spectra were obtained in the range of wavenumber 600–4000 m^−1^ (Figure 5A). ATR-IR spectra of the three films show all the main characteristic infrared absorption peaks of polyethylene terephthalate such as stretching vibration at 1714 cm^−1^ attributed to the carbonyl in the ester group and stretching vibrations of the ester groups at 1240 cm^−1^. Moreover, the stretching vibrations of the oxyethylene groups are indicated at 1120 cm^−1^ and vibration attributed to the group ν(O−CH_2_) and ν(C−C−C) are located at 1018 cm^−1^. The band at 724 cm^−1^ is specific for the vibration of the groups ν C(O)−O (r(C = O) and δ (CCO) assigned to the bending vibration out of the plane of the aromatic ring which provides information about the alignment of the chains in the polymer [43,46]. Moreover, peaks at 1340 and 1470 cm^−1^ are assigned to the trans-ethylene glycol conformer residue. This conformation of ethylene glycol is predominantly located in the crystalline phase of the film and its intensity is related to the increase of this conformer in the external layers of the PET films with respect to the bulk [42,47]. The IR results indicated a small increase in absorbance of the carbonyl region (1650–1750 cm^−1^) for plasma treated PET films (Figure 5B), which is in agreement with XPS results. However, there are not any statistically significant differences between peak areas of control, FLAIR^®^ and corona (Figure 5C). Incidentally, the thickness of the material contributing to the ATR-IR signal is only ~1–4 µm [48] but XPS as a highly sensitive surface analysis method probes the top 5–7 nm of the film [49]. In ATR-IR, the thickness of the sample cannot be uniquely determined for the whole spectrum and depends on the wavelength. This result shows that the plasma treatment does not change the chemical nature of the PET film in depth. Therefore, based on XPS and ATR-IR results, the surface modification occurs only in a nanometre thickness of the top layer of the film.

## 4. Conclusions

X-ray ptychography imaging using photon pulses from the soft X-ray free-electron laser FLASH was used for the first time to study the surface morphology of industrially relevant samples, i.e., the effects of various plasma treatments on the surfaces of PET films. Using X-ray ptychography, it was possible to reconstruct surface images that showed very good agreement with SEM images. Both techniques are capable of capturing the changes in surface morphology generated by the two plasma treatment techniques studied. X-ray ptychography is able to image a larger surface area and provides more reliable quantitative information on the three-dimensional morphology of the surface, such as the increase of the RMS surface roughness in a larger landscape. In summary, X-ray ptychography imaging shows great potential as an additional and complementary method to laboratory techniques such as AFM and SEM for imaging large surfaces of materials. The high-energy per treatment area was used to increase the density of the morphological features throughout the entire surface. However, the sensitivity of ptychography to variations of the sample thickness is independent of the treatment energy and defined by the refractive index of the sample. The spatial resolution of the ptychography technique is determined by the highest scattering angle detected [50]. Moreover, the sensitivity of ptychography can be further increased by switching to a reflective geometry, potentially achieving sub-nm profile sensitivity with nm spatial resolution [51,52]. Improvements to the experimental setup, especially an imaging detector with a higher dynamic range and frame rate, will allow a further improved resolution and reduced imaging time in the near future. A comparison of the two plasma treatment techniques leads to the conclusion that corona treatment significantly changes the morphology of the surfaces as seen from the imaging methods. On the other hand, the XPS results show that FLAIR^®^ treatments chemically alter the PET surface to a much larger extent by increasing the oxygen and nitrogen content and the polarity of the surface without significantly damaging the polymer surface, which is important for the printing and coating process. However, these changes were not observed in the ATR-IR results due to the lower sensitivity of the technique. Conclusively, the results indicate that the combination of X-ray ptychography and XPS can be used as a versatile toolbox for studying surface engineering and polymer film modifications.

## Figures and Tables

**Table 1:** Roughness parameters root-mean-square (*RMS*) roughness *R_q_*, interfacial development *S_dr_*, kurtosis *R_ku_* and skewness *R_sk_* of plasma-treated and untreated control PET films.

Roughness Parameters	Corona (5 W cm^−2^ & 3.6 s)	FLAIR^®^ (200 W cm^−2^ & 0.09 s)	Control
*R_q_* (nm)	20.98 ± 1.12	7.07 ± 1.01	3.83 ± 0.86
*S_dr_*	0.45 ± 0.03	0.05 ± 0.004	0.02 ± 0.004
*R_ku_*	2.97 ± 0.67	18.41 ± 0.98	8.29 ± 0.25
*R_sk_*	−0.75 ± 0.05	−2.76 ± 0.02	−1.68 ± 0.09

## Data Availability

Data will be made available on reasonable request.

## Supplementary Materials

The following supporting information can be downloaded at: https://www.mdpi.com/article/10.3390/polym14132528/s1, Figure S1: XPS spectra (counts per second (CPS) vs. binding energy (B.E.), survey and C 1s for (A) control PET film, (B) corona treated PET film, and (C) FLAIR treated PET film.
