# Supplementary material for: X-ray Ptychographic Imaging and Spectroscopic Studies of Plasma-Treated Plastic Films
Source: Polymers (Basel). 2022 Jun 21;14(13):2528. doi: 10.3390/polym14132528 (PMC9269290; doi:10.3390/polym14132528)
Supplement: Supplementary file 1 [file polymers-14-02528-s001.zip › polymers-1757769-supplementary.pdf]

X-ray ptychographic imaging and spectroscopic studies of plasma-treated plastic films

Mehdi Ravandeh[1], Masoud Mehrjoo*[2], Konstantin Kharitonov[2], Jan Schäfer[1], Antje Quade[1], Bruno Honnorat[1], Mabel Ruiz-Lopez[2], Barbara Keitel[2], Svea Kreis[2], Rui Pan[2], Seung-gi Gang[2], Kristian Wende*[1], Elke Plönjes[2]

[1]Leibniz Institute for Plasma Science and Technology, Felix-Hausdorff-Straße 2, 17489 Greifswald, Germany
[2]Deutsches Elektronen-Synchrotron DESY, Notkestraße 85, 22607 Hamburg, Germany

Supplementary information

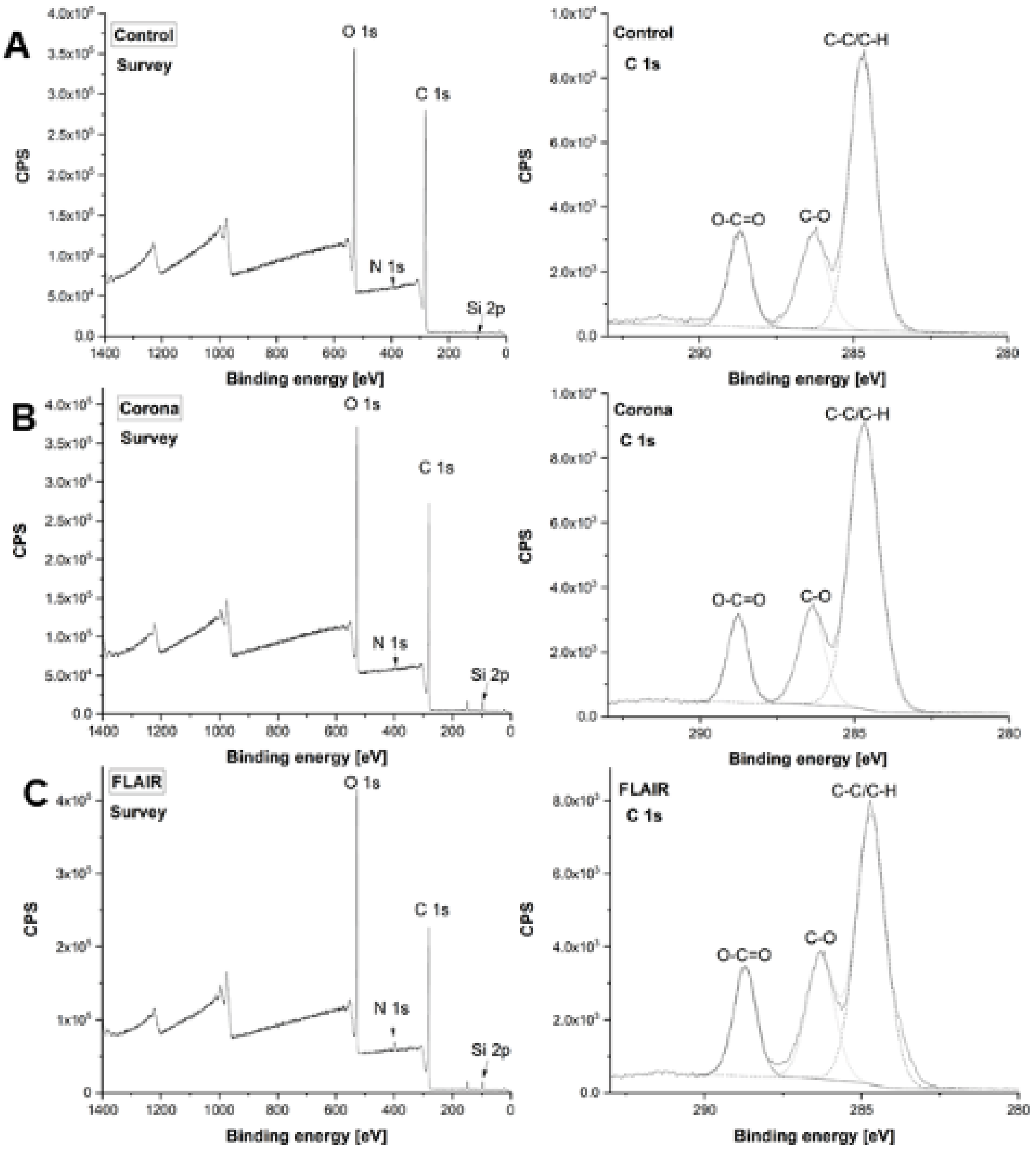

Figure S1. XPS Spectra (counts per second (CPS) vs. binding energy, B.E.), survey and C 1s for (A) control PET film, (B) corona treated PET film, and (C) FLAIR treated PET film
